# The complete mitochondrial genome of wild honeybee *Apis florea* (Hymenoptera: Apidae) in south-western China

**DOI:** 10.1080/23802359.2017.1407690

**Published:** 2017-11-25

**Authors:** Jie Yang, Jianxin Xu, Shaoyu He, Jie Wu

**Affiliations:** a Zhanjiang Experimental Station, Chinese Academy of Tropical Agricultural Sciences, Zhanjiang, China;; b Eastern Bee Research Institute, Yunnan Agricultural University, Kunming, China;; c Key Laboratory for Insect-Pollinator Biology of the Ministry of Agriculture, Institute of Apiculture, Chinese Academy of Agricultural Sciences, Beijing, China

**Keywords:** *Apis florea*, mitochondrial genome, phylogenetic analysis

## Abstract

We sequenced almost complete mitochondrial genome of *Apis florea* (Insecta: Hymenoptera: Apocrita: Apidae) with length of 15,933 bp. The genome has similar codon usage and gene organization to those of mitogenome reported for other Hymenoptera. It includes 13 protein-coding genes, two ribosomal RNA genes, 22 transfer RNA genes and a noncoding region with very high AT-base content. Phylogenetic analyses showed that, *Apis florea* and (*Apis cerana*+* Apis mellifera*) are the sister taxa, and more original, which is widely accepted view.

In this study, we presented the mitochondrial genome of the *Apis* species, *Apis florea*, which belong to corbiculate tribes. The species is extending some 7000 km from its eastern-most extreme in Vietnam and south-eastern China, across mainland Asia, along and below the southern flanks of the Himalayas, westwards to the Plateau of Iran and southwesterly into Oman (Hepburn et al. 2005). Specimen in this study was collected on 22 May 2010 in Meng Yang town, Jing Hong City, Yunnan province, southwest China (coordinate as follows: N22°04.070′E100°56.303′), and the specimen is stored in Kunming, Yunnan, China. The almost entire mitochondrial genome of *Apis florea* was 15,993 bp and deposited in the GenBank under the accession number KC170303 in NCBI. The genome has similar codon usage and gene organization to those of mitogenome reported for other Hymenoptera. It includes 13 protein-coding genes, two ribosomal RNA genes, 22 transfer RNA genes and a noncoding region with very high AT-base content. Genetic DNA from honeybee head was individually extracted. Annotation of the complete assembled mitochondrial genome was performed with Dual OrganellarGenoMe Annotator (DOGMA) (Wyman et al. 2004). The total base composition was A (43.46%), T (41.87%), G (5.48%), C (9.19%), suggesting that the percentage of A + T (85.33%) was higher than G + C (14.67%).

The phylogenetic analyses in this study concur with the proposed and widely accepted view of the internal relationships of *Apis*, *A. cerana* and *A. mellifera* are sister taxa and *A. florea* are more original (Arias and Sheppard 1996, 2005; Ratnieks 2006; Raffiudin and Crozier 2007). The consensus tree topology showed corbiculate bees to be a completely monophyletic taxa (Figure 1) and the relation between tribes was observed having 1000 bootstrap value. The complete mitochondrial genomes of seven other species are available from GenBank and the science name and accession numbers are as follows: *Apis cerana* (GQ162109), *Apis mellifera ligustica* (L06178), *Melipona bicolour* (NC_004529), *Bombus hypocrita sapporensis* (NC_011923), *Bombus ignites* (DQ870926), *Evania appendigaster* (NC_013238).

**Figure 1.**
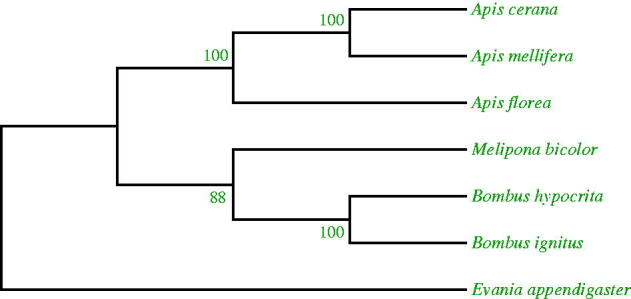
Inferred phylogenetic relationship among corbiculate tribes.
